# Cost-effectiveness of malaria diagnosis using rapid diagnostic tests compared to microscopy or clinical symptoms alone in Afghanistan

**DOI:** 10.1186/s12936-015-0696-1

**Published:** 2015-05-28

**Authors:** Kristian S Hansen, Eleanor Grieve, Amy Mikhail, Ismail Mayan, Nader Mohammed, Mohammed Anwar, Sayed H Baktash, Thomas L Drake, Christopher J M Whitty, Mark W Rowland, Toby J Leslie

**Affiliations:** Department of Global Health and Development, London School of Hygiene & Tropical Medicine, 15-17 Tavistock Place, London, WC1H 9SH UK; Health Economics and Health Technology Assessment, Institute of Health and Wellbeing, University of Glasgow, Glasgow, UK; Department of Disease Control, London School of Hygiene & Tropical Medicine, Keppel Street, London, WC1E 7HT UK; Health Protection and Research Organization, Kabul, Afghanistan; HealthNet-TPO, Kabul, Afghanistan; Merlin, Kabul, Afghanistan; Department of Clinical Research, London School of Hygiene & Tropical Medicine, Keppel Street, London, WC1E 7HT, UK

**Keywords:** Cost-effectiveness analysis, Malaria, Rapid diagnostic test, Microscopy diagnosis, Clinical diagnosis, *Plasmodium vivax*, *Plasmodium falciparum*, Afghanistan

## Abstract

**Background:**

Improving access to parasitological diagnosis of malaria is a central strategy for control and elimination of the disease. Malaria rapid diagnostic tests (RDTs) are relatively easy to perform and could be used in primary level clinics to increase coverage of diagnostics and improve treatment of malaria.

**Methods:**

A cost-effectiveness analysis was undertaken of RDT-based diagnosis in public health sector facilities in Afghanistan comparing the societal and health sector costs of RDTs *versus* microscopy and RDTs *versus* clinical diagnosis in low and moderate transmission areas. The effect measure was ‘appropriate treatment for malaria’ defined using a reference diagnosis. Effects were obtained from a recent trial of RDTs in 22 public health centres with cost data collected directly from health centres and from patients enrolled in the trial. Decision models were used to compare the cost of RDT diagnosis *versus* the current diagnostic method in use at the clinic per appropriately treated case (incremental cost-effectiveness ratio, ICER).

**Results:**

RDT diagnosis of *Plasmodium vivax* and *Plasmodium falciparum* malaria in patients with uncomplicated febrile illness had higher effectiveness and lower cost compared to microscopy and was cost-effective across the moderate and low transmission settings. RDTs remained cost-effective when microscopy was used for other clinical purposes. In the low transmission setting, RDTs were much more effective than clinical diagnosis (65.2% (212/325) *vs* 12.5% (40/321)) but at an additional cost (ICER) of US$4.5 per appropriately treated patient including a health sector cost (ICER) of US$2.5 and household cost of US$2.0. Sensitivity analysis, which varied drug costs, indicated that RDTs would remain cost-effective if artemisinin combination therapy was used for treating both *P. vivax* and *P. falciparum*. Cost-effectiveness of microscopy relative to RDT is further reduced if the former is used exclusively for malaria diagnosis. In the health service setting of Afghanistan, RDTs are a cost-effective intervention compared to microscopy.

**Conclusions:**

RDTs remain cost-effective across a range of drug costs and if microscopy is used for a range of diagnostic services. RDTs have significant advantages over clinical diagnosis with minor increases in the cost of service provision.

**Trial Registration:**

The trial was registered at ClinicalTrials.gov under identifier NCT00935688.

**Electronic supplementary material:**

The online version of this article (doi:10.1186/s12936-015-0696-1) contains supplementary material, which is available to authorized users.

## Background

Improving access to parasitological malaria diagnosis is a central strategy for control and elimination of the disease and is recommended before anti-malarial treatment in all suspected malaria cases [1,2]. Outside most of sub-Saharan Africa, diagnosis needs to differentiate between *Plasmodium vivax* and *Plasmodium falciparum* because treatment differs between the two species. Diagnosis in most primary level clinics in south and west Asia relies either on symptoms and signs alone or on microscopy [3,4]. Both of these methods have significant drawbacks: symptoms and signs are indistinguishable from other causes of fever [5] and cannot differentiate between species, while microscopy is often inaccurate under field conditions, hard to maintain, requires skilled staff and can suffer from a tendency by health providers to treat patients with negative test results [6-10].

Rapid diagnostic tests (RDTs) for malaria are a potential alternative to both clinical and microscopy-based diagnosis since the former are easier to perform, require limited training and have high accuracy both under controlled and field conditions [2,11-13]. Research from different settings in Africa and Asia suggests that RDTs have a significant advantage over presumptive diagnosis and equal or better performance than light microscopy under field conditions [3,9,14-16]. Almost all studies indicate that RDTs improve appropriate malaria treatment (interpreted as prescribing anti-malarials only to those patients with malaria parasites) when compared to presumptive diagnosis. However, the advantage of RDTs over microscope diagnosis is typically smaller or insignificant [17,18]. Fewer studies on operational issues of malaria diagnostics have been performed in west and south Asia where in excess of 1.5 billion people live in areas of malaria risk, and vivax malaria is the predominant species. In Afghanistan, the introduction of malaria RDTs with relatively simple training in lower level health centres was found to increase the proportion of patients appropriately treated for malaria as compared to presumptive diagnosis and microscopy [3]. The effect was particularly strong for the detection and treatment of cases of *P. falciparum* malaria when relatively rare.

An important consideration for selecting malaria diagnostic methods within health systems is the comparative cost-effectiveness. Assessment of cost-effectiveness provides data on the costs and sustainability of programmes, which is particularly important to policy makers when countries are considering elimination of malaria; in the later stages of elimination, very few malaria cases will be found, but facilities must continue to provide malaria diagnosis to maintain suitable levels of surveillance.

Analyses from African settings suggest that the introduction of malaria diagnosis by falciparum-specific RDT is likely to be a cost-effective intervention compared to presumptive diagnosis [19-23], while the cost-effectiveness advantage of RDTs over microscopy tends to be smaller or insignificant [23-27]. However, cost-effectiveness depends critically on a range of factors, including malaria transmission intensity, health workers’ adherence to test results when providing treatment, and the underlying costs and accuracy of the parasitological tests [19,20,22,24-26].

Most of these factors differ between Africa (where the majority of studies have been undertaken) and south and central Asia where no studies have examined the cost-effectiveness of malaria diagnostics. In particular, malaria transmission is generally much lower in south and central Asia and *P. vivax* is the dominant species [4]. This increases the cost of tests, which have to be able to detect two species, whilst reducing the number of true cases. Only a few studies have been conducted in areas endemic for both *P. falciparum* and *P. vivax*, in Southeast Asia and the Pacific. A cost-effectiveness analysis was undertaken to assess the cost-effectiveness of current diagnostic methods (presumptive or microscopy) with RDT diagnosis in two different epidemiological settings within the context of a randomized, controlled trial in Afghanistan [3].

### Ethical approval

The trial protocol was approved by the Institutional Review Board of the Ministry of Public Health, Islamic Republic of Afghanistan (No. 112453) and by the ethics review board of the London School of Hygiene and Tropical Medicine (No. 5386).

## Methods

Cost-effectiveness analyses of malaria diagnostic methods from a health sector and societal perspective were conducted in two regions of Afghanistan with different transmission levels. The purpose of these analyses was to determine if diagnosis by RDT would be a better use of scarce resources than the malaria diagnostic methods currently in use in the public health care sector in Afghanistan. In order to investigate this purpose, incremental cost-effectiveness analysis was used, which by definition is the ratio between the difference in cost if a new health intervention replaces the intervention currently in place and the difference in effect when the current intervention is replaced by the new.

Currently, the health sector relies on microscopy and clinical diagnosis. Incremental cost-effectiveness analyses were performed for replacing microscopy diagnosis with RDT and also for introducing RDT diagnosis instead of clinical diagnosis. These incremental cost-effectiveness analyses were undertaken using a decision model where all necessary inputs on costs, effects and probabilities were obtained from data collection conducted in Afghanistan during 2009–2012.

### Study setting

Malaria transmission in Afghanistan varies considerably by area ranging from moderate to very low. *Plasmodium vivax* is by far the most common species accounting for at least 90% of malaria cases annually while *P. falciparum* is responsible for the remaining malaria infections [4]. For the trial, one moderate transmission province in the east adjoining Pakistan and a low transmission province in the north bordering Tajikistan were chosen. Three distinct malaria diagnostic settings were identified: (1) In the moderate transmission province, light microscopy had been a routine component of the services in public health centres for many years and microscopists performed malaria microscopy as well as a range of other diagnostic tests; (2) in the low transmission province, new light microscopes had recently been introduced in some health centres with the focus exclusively on providing malaria parasitological diagnosis and at the time of the study, the microscopes were not used for any other diagnostic investigations; and, (3) the remaining health centres in the same low transmission area did not have any microscopy capabilities so clinicians diagnosed malaria based on clinical signs and symptoms alone.

According to national treatment guidelines patients with microscopy or RDT confirmed *Plasmodium vivax* receive chloroquine (25 mg/kg in divided doses over 3 days); with *P. falciparum* or mixed infections receive artemisinin combination therapy (4 mg/kg/day artesunate for 3 days and a single administration of 25/1.25 mg/kg sulfadoxine-pyrimethamine on day 1); and clinically diagnosed patients with suspected malaria receive combination therapy with sulfadoxine-pyrimethamine (25/1.25 mg/kg on day 0) and chloroquine (10 mg/kg on days 0 and 1 and 5 mg/kg on day 2).

### Interventions

The intervention assessed in this study was the introduction of malaria RDTs which were able to distinguish *P. falciparum* from other *Plasmodium* species (AccessBio CareStart malaria RDT Pf (HRPII)/Pan (pLDH)) compared to standard care in each setting as described above. A total of 12 health centres with long-established microscopy were selected for this study from the moderate transmission area, while ten health centres from the low transmission area were chosen, five of which had newly established microscopy focusing on malaria only while the other five used presumptive malaria diagnosis. The clinics were purposively selected if they were in secure and accessible areas, and treated malaria or suspected malaria cases. The clinics were typical of the Afghan health system’s lower tiers defined as Basic Health Centres or Comprehensive Health Centres [28,29] and were overseen and managed by non-governmental organisations.

### Measurement of effect

Suspected malaria patients visiting study health centres and giving consent to participate in this study were randomized to either a RDT or the currently used diagnostic method at the particular health centre. Between September 2009 and September 2010, a total of 5,749 patients were enrolled [3]. The measure of effect for the diagnostic interventions was appropriate treatment of suspected malaria. Appropriate treatment was assessed against a reference diagnosis and matched the national malaria treatment guidelines. This was defined as patients with *P. vivax* prescribed with chloroquine, patients with *P. falciparum* prescribed with artemisinin combination therapy (ACT) and malaria negative patients not receiving an anti-malarial. The reference diagnosis was based on PCR-confirmed, double-read thick and thin Giemsa-stained blood slides from all patients enrolled in the trial [3]. The main results, describing full details of the trial, including the effect of each diagnostic technique in the different settings on appropriate treatment of malaria have been published previously [3]. Results are summarized in Table 1.

**Table 1:** **Effects of malaria diagnostic methods on appropriately treated patients in two regions of Afghanistan, 2009**

	**Moderate transmission region**		**Low transmission region**			
			**Area with microscopes**		**Area without microscopes**	
**Effects**	**Microscopy arm**	**RDT arm**	**Microscopy arm**	**RDT arm**	**Clinical arm**	**RDT arm**
Fever patients suspected of malaria (N)	1,983	2,028	515	523	321	325
Of which suffering from (%)^#^:						
vivax malaria	21.9	19.5	1.0	0.4	0.0	0.0
falciparum malaria	3.8	3.4	0.0	0.0	0.0	0.0
Mixed vivax-falciparum infections	0.1	0.1	0.0	0.0	0.0	0.0
Non-malarial febrile illness	74.3	77.0	99.0	99.6	100.0	100.0
Effect:						
Patients appropriately treated (N)	1,512	1,696	393	420	40	212
Patients appropriately treated (%)	76.2	83.6	76.3	80.3	12.5	65.2
Adjusted^&^ odds ratio	1	1.70^**^	1	1.73^*^	1	92.7^**^
(95% CI)	(1.30; 2.23)		(1.08; 2.78)			(12.4; 694.1)

^#^ According to the malaria reference diagnosis.

^&^ Adjusted odds ratio using three level model, adjusted for fixed effect of patient’s age and clinic type (in moderate transmission region) and random effects of clinician (within clinics) and clinic (between clinics).

^*^ p < 0.05, ^**^ p < 0.001.

Source: [3].

### Measurement of cost

Costs per service in the public sector for a diagnosis by microscopy and RDT and treatment for malaria and non-malarial febrile illnesses were estimated based on data collected from the non-governmental organizations that managed the health centres in the study area in 2010. Three health centres in the moderate transmission east region were excluded due to security problems and/or gaps in the data collected. At each of the remaining 19 health centres, total recurrent expenditure for the financial year 2009 were obtained incorporating cost categories, including salaries of all personnel, medicines, consumables, utilities, maintenance, cleaning materials, domestic expenses, etc. Construction cost of health centre buildings was estimated by obtaining a plan of a typical basic and comprehensive health centre in Afghanistan as well as construction cost per square metre from the non-governmental organizations. An inventory of equipment and furniture by room in the health centres was done and each item was subsequently valued by their 2009 purchase prices. Since buildings, equipment and furniture had a useful lifespan longer than one year, equivalent annual cost of the total costs of these inputs were estimated using a standard annualization procedure with a discount rate of 0.03 and expected life spans of 20 years for buildings and seven years for equipment and furniture [30].

These aggregate health centre level costs for 2009 were then allocated to patient services offered at the health centres using the standard step-down costing method [30,31]. This method allocates aggregate cost by category to overhead and intermediate services and finally to patient services in a step-wise fashion using allocation criteria reflecting actual resource use. For instance, total salary cost was allocated to relevant services based on discussions with key personnel on how they spend a typical day across services.

The step-down costing method was supplemented by micro-costing methods [32,33] in order to separate out specifically the cost of services relevant for this study, including malaria microscopy and RDT diagnosis in the laboratories (or dedicated rooms) and treatment of malaria and non-malaria fevers in the treatment rooms. A laboratory scientist (AM) specified the types and amounts of reagents and disposables required to perform one malaria blood slide and similarly for an RDT. For the health centres in the moderate transmission eastern region, the annual equivalent cost of the microscope was divided between malaria slide diagnostics and other microscope-based tests according to relative use. In the low transmission northern region, the microscope was only used for investigating malaria blood smears so the whole annual equivalent cost of a microscope was allocated to malaria microscopy. Microscopists in the health centres were asked to estimate the time required to prepare a malaria slide followed by searching for parasites using the microscope and to perform an RDT, respectively. In some of the health centres, the activity data collected suggested that microscopists on average had time available after having finalized the tests required for a day. In these health centres, the personnel time and consequently the cost per test was adjusted upwards to capture this opportunity cost of available time.

Malaria and non-malarial febrile illnesses were treated in the consultation room of the health centre. Assuming that clinicians generally followed official clinical guidelines, the doses for treatment of a child and an adult with malaria specified in these guidelines and prices from medical stores in Afghanistan were used to estimate the cost per course of ACT (artesunate/sulphadoxine-pyrimethamine (SP)) for treating falciparum malaria and chloroquine (and in some instances including SP) for treating vivax malaria. Cost per course of antibiotic treatment for non-malarial febrile illnesses was calculated using the same method. These drug costs were adjusted upwards to include the cost of running the pharmacy/dispensary at the health centres, which had been captured as part of the standard step-down costing methodology. Finally, doctors and nurses working in the consultation rooms were asked to estimate the required average time used for an outpatient department visit, including taking illness history and prescribing drugs, which were subsequently utilized for estimating salary cost per patient visit based on salary level by personnel category.

The combination of the step-down and micro-costing methodologies enabled the estimation of a comprehensive cost per service in 19 health centres by malaria diagnostic method and treatment incorporating both immediate resources such as drugs, disposables and personnel time, but also shared resources such as administration of the health centre, security, buildings and equipment. The results of the health services costing exercise are presented separately for each region in Tables 2 and 3.

**Table 2:** **Cost per unit of health service in study health centres and household cost per treatment seeking episode, moderate transmission region of Afghanistan, cost in US$ (AFN50.33 = US$1), 2009**

	**Mean**	**Median**	**Minimum**	**Maximum**	**IQR**
*Health sector cost per service in US$* ^*#*^					
Microscopy	1.89	1.87	1.61	2.21	
Rapid diagnostic test	1.92	1.97	1.63	2.23	
Outpatient visit excl drugs	0.47	0.43	0.37	0.64	
Artimisinin-based combination therapy (SP/AS), adult course	1.14	1.14	1.10	1.19	
Artimisinin-based combination therapy (SP/AS), child course	0.65	0.65	0.63	0.68	
Chloroquine, adult course	0.14	0.14	0.13	0.14	
Chloroquine, child course	0.06	0.06	0.06	0.07	
Sulphadoxine-pyrimethamine, adult course	0.14	0.14	0.13	0.14	
Sulphadoxine-pyrimethamine, child course	0.06	0.06	0.06	0.07	
Antibiotics, adult course	0.14	0.14	0.13	0.14	
Antibiotics, child course	0.14	0.14	0.13	0.14	
*Diagnostics training cost in US$ for health personnel per patient enrolled*					
Microscopy	0.10				
Rapid diagnostic test	0.03				
*Household cost in US$ (over a period of 28 days from the first visit) * ^&^					
Out-of-pocket expenditure, patient appropriately treated	3.77	0.00	0.00	129.15	(0.00; 0.00)
Out-of-pocket expenditure, patient inappropriately treated	6.92	0.00	0.00	116.11	(0.00; 1.64)
Opportunity cost of time lost, patient appropriately treated	1.71	0.00	0.00	25.93	(0.00; 2.47)
Opportunity cost of time lost, caregiver of patient appropriately treated	0.65	0.00	0.00	16.05	(0.00; 0.00)
Opportunity cost of time lost, patient inappropriately treated	2.67	0.00	0.00	19.75	(0.00; 3.70)
Opportunity cost of time lost, caregiver of patient inappropriately treated	0.98	0.00	0.00	9.88	(0.00; 1.23)

^#^ Incorporates the full cost of offering services at public health centres including prices of RDTs, microscopy consumables or drugs as well as personnel, dispensing, utilities, and capital cost of buildings and equipment.

^& ^Lost time valued at US$1.23 per day equal to GDP per capita per day in 2009 [34].

IQR = interquartile range.

**Table 3:** **Cost per unit of health service in study health centres and household cost per treatment seeking episode, low transmission region of Afghanistan, cost in US$ (AFN50.33 = US$1), 2009**

	**Mean**	**Median**	**Minimum**	**Maximum**	**IQR**
*Health sector cost per service in US$* ^*#*^					
Microscopy	8.32	7.99	6.92	9.89	
Rapid diagnostic test	1.30	1.31	1.28	1.33	
Outpatient visit excl drugs	0.37	0.40	0.26	0.45	
Artimisinin-based combination therapy (SP/AS), adult course	1.28	1.28	1.21	1.42	
Artimisinin-based combination therapy (SP/AS), child course	0.73	0.73	0.69	0.81	
Chloroquine, adult course	0.16	0.15	0.15	0.17	
Chloroquine, child course	0.07	0.07	0.07	0.08	
Sulphadoxine-pyrimethamine, adult course	0.16	0.15	0.15	0.17	
Sulphadoxine-pyrimethamine, child course	0.07	0.07	0.07	0.08	
Antibiotics, adult course	0.16	0.15	0.15	0.17	
Antibiotics, child course	0.16	0.15	0.15	0.17	
*Diagnostics training cost in US$ for health personnel per patient enrolled*					
Microscopy	0.10				
Rapid diagnostic test	0.03				
*Household cost in US$ (over a period of 28 days from the first visit) * ^&^					
Out-of-pocket expenditure, patient appropriately treated	3.12	0.00	0.00	38.74	(0.00; 1.99)
Out-of-pocket expenditure, patient inappropriately treated	2.26	0.00	0.00	21.46	(0.00; 2.78)
Opportunity cost of time lost, patient appropriately treated	5.80	4.94	0.00	19.75	(1.23; 8.64)
Opportunity cost of time lost, caregiver of patient inappropriately treated	3.20	2.47	0.00	11.11	(0.00; 6.17)
Opportunity cost of time lost, patient inappropriately treated	5.20	3.70	0.00	12.35	(3.70; 8.64)
Opportunity cost of time lost, caregiver of patient inappropriately treated	2.73	3.70	0.00	8.64	(0.00; 3.70)

^# ^Incorporates the full cost of offering services at public health centres including prices of RDTs, microscopy consumables or drugs as well as personnel, dispensing, utilities, and capital cost of buildings and equipment.

^& ^Lost time valued at US$1.23 per day equal to GDP per capita per day in 2009 [34].

IQR = interquartile range.

Household cost of healthcare seeking was captured in a sample of 676 suspected malaria patients (508 in the moderate transmission and 168 in the low transmission region) for a 28-day period starting from the day a patient first sought care at one of study health centres. Patients from the main trial were randomly selected to participate in the household cost component which involved being interviewed on the day of the first visit to a study health centre and subsequently receiving a visit in their homes by a trained interviewer on day 7, day 14 and day 28 after the initial health centre visit. When leaving the health centre after the initial visit, these patients were interviewed to capture information on their out-of-pocket expenditure for transport, drugs, consultation fees, laboratory fees, or special foods incurred in relation to the present visit. The purpose of all the subsequent home visits was to find out from the patient if there had been any additional treatment seeking to any kind of health provider since the previous interview and if yes, information was captured on the same categories of out-of-pocket expenditure. In public sector clinics, no fees for consultation, drugs or diagnostics are paid so these costs were captured as part of the facility costing above. In addition, all interviews inquired if the fever illness had resulted in days where the patient and the main caregiver were unable to perform their normal activities and how much time had been spent travelling to and waiting at health providers. This opportunity cost of time lost was valued at GDP per capita per day in 2009 [34], which amounted to US$1.23 per day. Household costs are displayed in Tables 2 and 3.

Finally, the costs of RDT and microscopy training for health workers were included and classified as health sector cost (Tables 2 and 3). RDT training was minimal but realistic, to reflect feasibility of implementation at scale, and therefore expected to be a one-day workshop occurring every five years. Microscopy refresher training currently lasts three days and training is envisaged every three years within a standard quality assurance system. Refresher training costs included per diem and transport of trainers and participants, workshop materials and refreshments. Costs of the initial training of microscopists were not included.

### Decision analysis

**Figure 1:**
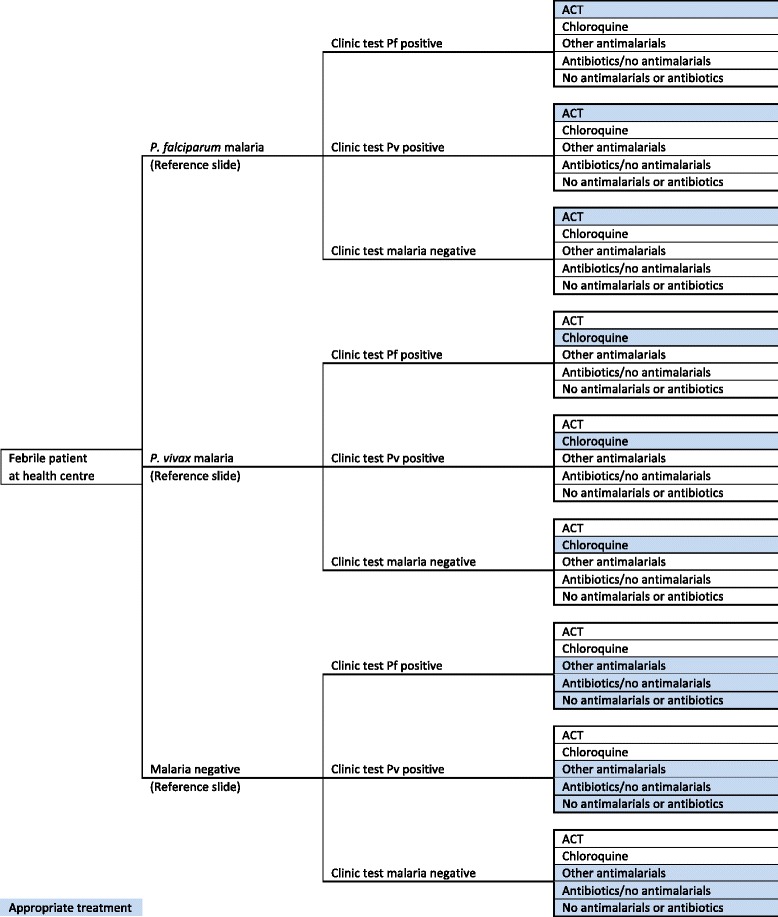
Decision tree for the malaria diagnostic method used in each of the study arms in two regions of Afghanistan, 2009.

A decision tree was constructed for the control and intervention arms in the three diagnostic settings to compare the different possible series of pathways leading to the trial’s primary endpoint of a febrile patient being appropriately treated (Figure 1). Arriving at a health centre, febrile patients either had malaria (*P. falciparum* or *P. vivax*) or a non-malarial febrile illness (NMFI) as determined by their reference diagnosis. Each patient was then given a blood test or clinically diagnosed at the health centre depending on study arm and diagnostic setting. Healthcare personnel either complied or did not comply with the diagnostic test result and subsequently decided on the medical treatment.

The probabilities assigned to each decision tree branch relating to malaria status according to reference slide, clinic diagnosis accuracy and drug prescription behaviour of health personnel were obtained from the trial [3] and are reproduced in Additional file 1. The mean costs of health services obtained from study health centres and household costs (Tables 2 and 3) were also assigned to the relevant branches of the trees. The expected costs and effects associated with each diagnostic test or clinical diagnosis were then calculated by ‘rolling back’ the decision tree along the branches. Thus, the incremental cost per additional febrile patient appropriately treated of the alternative option could be compared.

### Probabilistic sensitivity analysis

The uncertainty surrounding the cost-effectiveness results was assessed through probabilistic sensitivity analysis (PSA) [35-37]. Distributions were assigned to all relevant parameters in order to build into the decision model the combined implications of uncertainty surrounding each of the model inputs. Details are shown in Additional file 1. Probabilities can necessarily only take values between zero and one. Beta distributions were fitted when decision nodes had two branches. Other nodes had multiple branches, including diagnostic accuracy and treatment, and Dirichlet distributions were fitted to ensure that mutually exclusive event probabilities summed to one [38]. Gamma distributions were fitted to household costs given the skewed nature of the data [35]. Monte Carlo simulation was used to propagate uncertainty by randomly selecting values from each parameter’s distribution by running 1,000 iterations. The generated results of expected outcomes for RDT *versus* current diagnostic method were expressed as monetary net benefits for a specified value of willingness-to-pay (λ), the option with the higher net benefits being the cost-effective for any given set of randomly drawn input values. The model was run for values of λ from $0 to $100 (in steps of $5). The resultant output for each trial setting, i.e., the probability of how often RDT or usual care is the cost-effective intervention for each value of λ, is presented graphically as a cost-effectiveness acceptability curve [39,40].

### Scenario analysis

The context of extremely low prevalence in the northern region of the study area (less than 1%) offered an opportunity to estimate the incremental costs of selected malaria elimination efforts. In the northern region of the study area, new microscopes and trained technicians were introduced specifically to contribute to the elimination of malaria in the area. The decision model was used to estimate the cost per malaria case appropriately treated in the three settings compared with blanket coverage of treatment with paracetamol (an antipyretic drug) alongside no parasitological testing. Only costs from a health sector perspective were included. In a second scenario analysis, the model was used to examine the replacement of chloroquine (used for the treatment of vivax malaria) and ACT for treatment of falciparum malaria, with ACT used for treating both vivax and falciparum malaria. Published trial reports of ACT *versus* chloroquine to treat vivax show that vivax infections treated with SP/artesunate (AS) or other ACTs would be as good, or potentially superior to the standard treatment with chloroquine alone [41,42]. This scenario was considered because treating all malaria with ACT may simplify treatment protocols for health workers and de-emphasize the importance of high quality, differentiated diagnosis. Microscopists can accurately identify malaria parasites on blood smears, but it is less easy to ensure correct species identification in low-endemic settings with low parasite density infections [3,43]. Currently, SP/AS is the recommended ACT in Afghanistan but this drug may be replaced in the future if SP resistance is demonstrated through molecular or *in vivo* monitoring. An attractive alternative is dihydroartemisinin-piperaquine (DHA-PPQ) [42], but this drug combination is considerably more expensive. The decision model was therefore used to also assess the impact of drug price variation by modelling the use of more expensive ACT for treatment of both *P. vivax* and *P. falciparum*.

## Results

The results of the probabilistic cost-effectiveness analyses are presented in Table 4. In the moderate transmission eastern region, the societal cost per patient was marginally lower in the RDT arm compared to the microscopy arm (US$9.5 *versus* US$9.8) but the effect in terms of appropriately treated patients was higher in the RDT arm than in the microscopy arm (83.7%, CI: (82.0%; 85.3%) *versus* 76.3%, CI: (74.4%; 78.2%)). Since the effect was higher and the cost was lower, diagnosis by RDT is the dominant option compared to diagnosis by microscopy from an economic perspective. In the low transmission northern region with recently installed new microscopes in health centres, the societal cost per patient was higher in the microscopy arm compared with the RDT arm (US$20.6 *versus* US$13.5). The effect in terms of the number of appropriately treated patients was slightly higher in the RDT arm (80.2%, CI: (77.0%; 83.7%)) compared to the microscopy arm (76.2%, CI: (72.4%; 80.0%)). Diagnosis by RDT was therefore dominant from an economic perspective in the low transmission area. Finally, in the low transmission northern region where the remaining health centres did not have microscopes and relied on presumptive diagnosis, the effect as measured by the proportion of appropriately treated patients was much higher when diagnosing by RDT as compared to presumptive diagnosis (65.9%, CI: (60.6%; 71.1) *versus* 12.5%, CI: (9.1%; 16.1%)). The societal cost per patient was slightly higher when using RDT diagnosis compared to presumptive diagnosis (US$13.2 *versus* US$10.8) leading to an incremental cost-effectiveness ratio (ICER) of US$4.5 per appropriately treated patient.

**Table 4:** **Cost per patient in US$, effects and incremental cost-effectiveness ratios (ICERs) of replacing current diagnostic methods by rapid diagnostic tests in two regions of Afghanistan, cost in US$ (AFN50.33 = US$1), 2009**

	**Moderate transmission region**		**Low transmission region**			
			**Area with microscopes**		**Area without microscopes**	
	**Microscopy arm**	**RDT arm**	**Microscopy arm**	**RDT arm**	**Clinical arm**	**RDT arm**
Appropriately treated patients in % (95% CI)	76.3 (74.4; 78.2)	83.7 (82.0; 85.3)	76.2 (72.4; 80.0)	80.2 (77.0; 83.7)	12.5 (9.1; 16.1)	65.9 (60.6; 71.1)
Cost per patient in US$ (95% CI):						
Health sector cost	2.6 (2.6; 2.6)	2.6 (2.5; 2.6)	9.0 (9.0; 9.0)	1.9 (1.8; 1.9)	0.5 (0.5; 0.5)	1.8 (1.8; 1.8)
Household cost	7.2 (0.1; 37.6)	6.9 (0.1; 36.0)	11.6 (3.6; 28.4)	11.6 (3.4; 29.5)	10.3 (3.4; 23.6)	11.4 (3.6; 25.7)
Total societal cost	9.8 (2.7; 40.0)	9.5 (2.6; 40.5)	20.6 (12.5; 36.4)	13.5 (5.2; 30.3)	10.8 (3.9; 23.8)	13.2 (5.7; 28.3)
Incremental analysis (95% CI):	Replace microscopy diagnosis by RDT		Replace microscopy diagnosis by RDT		Replace presumptive diagnosis by RDT	
Increase in patients appropriately treated in %	7.4 (5.0; 9.8)		4.0 (−0.8; 8.8)		53.4 (47.4; 59.5)	
Incremental cost in US$, health sector perspective	−0.0 (−0.0; −0.0)		−7.1 (−7.1; −7.1)		1.3 (1.3; 1.3)	
Incremental cost in US$, societal perspective	−0.3 (−4.4; 2.8)		−7.1 (−8.0; −6.0)		2.4 (−7.5; 15.7)	
ICER in US$, health sector perspective	Dominant		Dominant		2.5	
ICER in US$, societal perspective	Dominant		Dominant		4.5	

**Figure 2:**
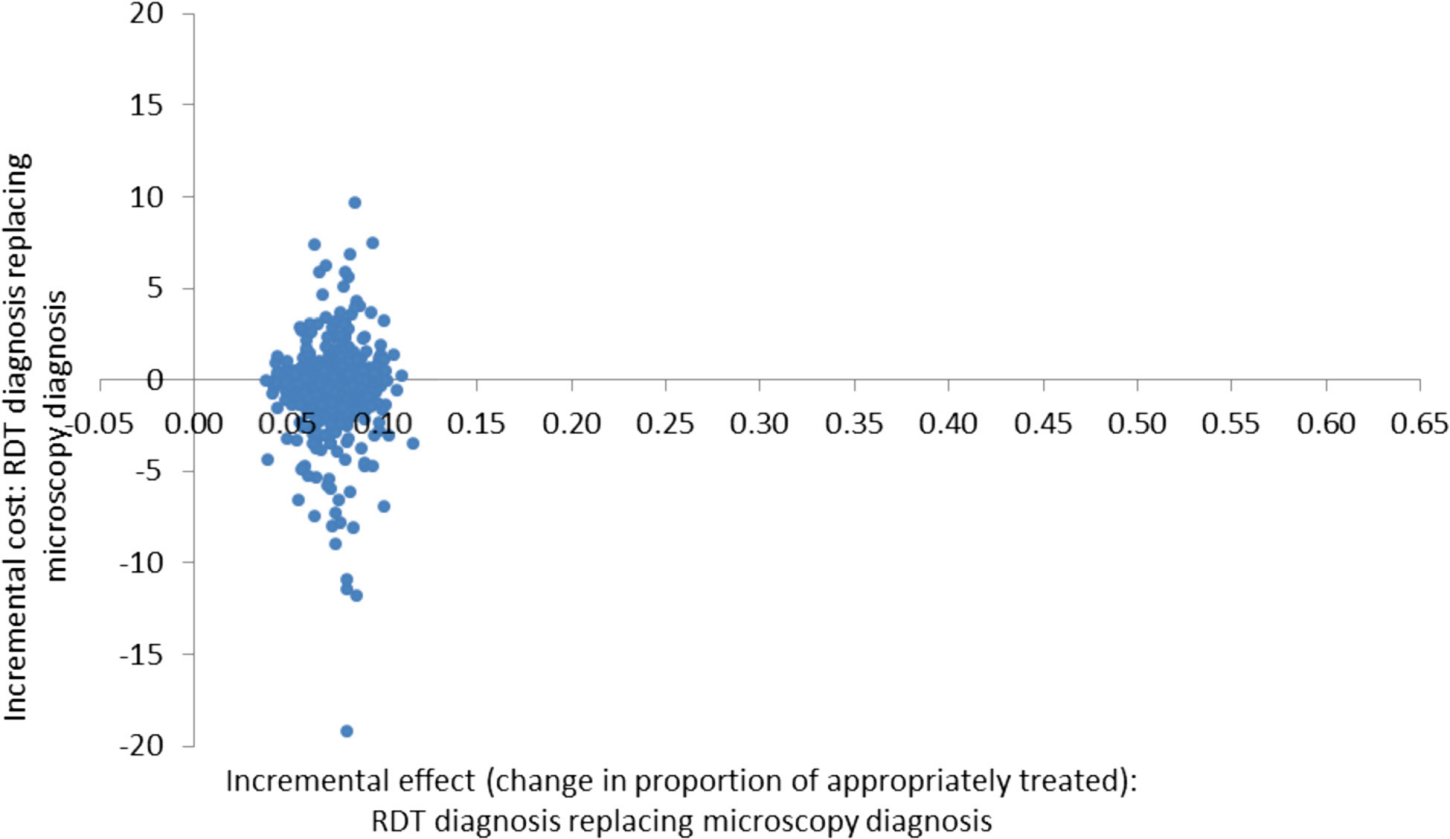
Cost-effectiveness plane of RDT compared with microscopy diagnosis in a moderate transmission region of Afghanistan, 2009: scatterplot of incremental societal costs in US$ and incremental effect of appropriately treated patients. Average incremental cost: −US$0.32, average incremental effect: 0.07.

**Figure 3:**
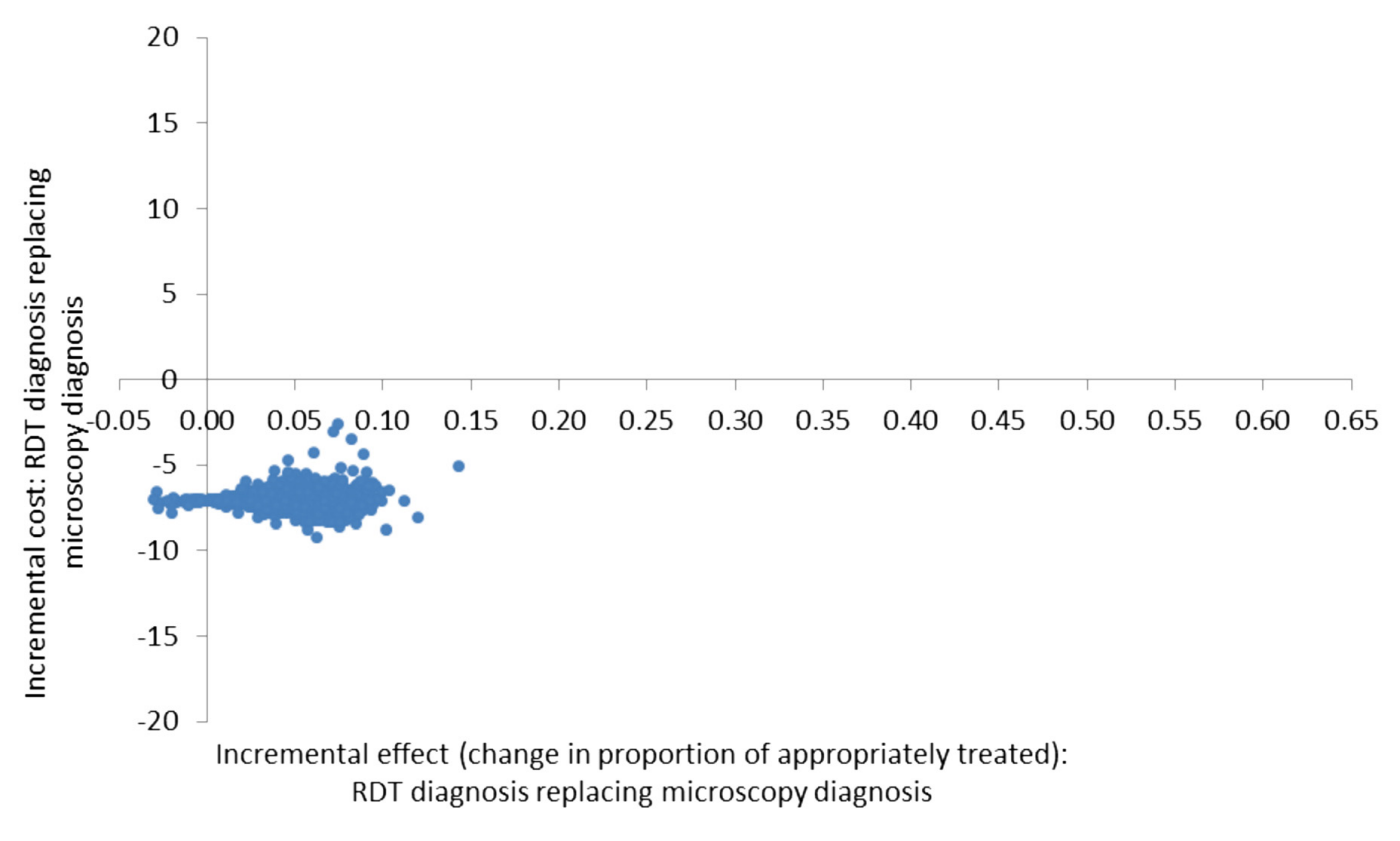
Cost-effectiveness plane of RDT diagnosis compared with microscopy diagnosis in a low transmission region of Afghanistan, 2009: scatterplot of incremental societal costs in US$ and incremental effect of appropriately treated patients. Average incremental cost: −US$7.05, average incremental effect: 0.04.

**Figure 4:**
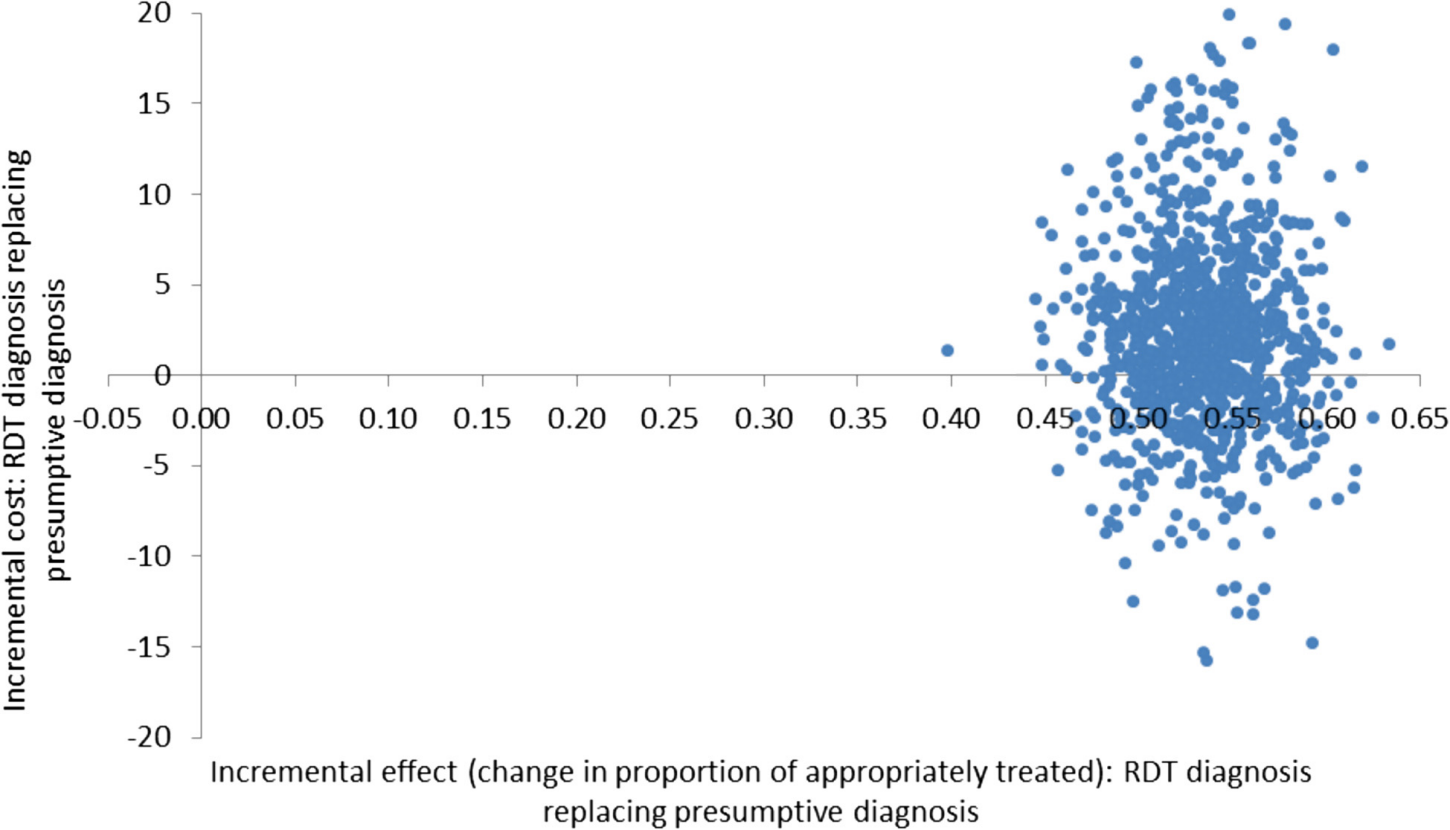
Cost-effectiveness plane of RDT compared with presumptive diagnosis in a low transmission region of Afghanistan, 2009: scatterplot of incremental societal costs in US$ and incremental effect of appropriately treated patients. Average incremental cost: US$2.38, average incremental effect: 0.53.

The cost-effectiveness planes (Figures 2, 3 and 4) show results from the PSA and identify the extent of the uncertainty in the incremental costs (y-axis) and in the incremental outcomes (x-axis). In the moderate transmission region where RDT diagnosis was compared to microscopy, estimates of the incremental effect are always positive, meaning that the percentage of appropriately treated patients is always higher when using an RDT compared to microscopy, with mean incremental effect of 7.4% (CI: 5.5%; 9.8%) (Figure 2). However, the incremental costs are both positive and negative, suggesting that there is uncertainty with regard to which diagnostic method has the highest cost per patient although, on average, RDT costs are lower with a mean incremental cost of -US$0.3 (CI: −US$4.4; US$2.8). In the low transmission area comparing RDT and microscopy diagnosis, incremental societal costs are always negative so that societal cost per patient in the RDT arm is always lower than in the microscopy arm with mean incremental cost of -US$7.1 (CI: −US$8.0; −US$6.0) (Figure 3). RDTs showed a better effect in terms of the number of appropriately treated patients in most iterations of the Monte Carlo simulation although in a minority of these, microscopy had a higher effect. The mean incremental effect was 4.0% (CI: −0.8%; 8.8%). While this suggested that no significant difference could be demonstrated between RDT and microscopy, adjusting for a range of factors including patient age and health centre characteristics did result in a significantly higher number of appropriately treated patients in the RDT arm (see Table 1). In the low transmission region where diagnosis was presumptive, the results of the PSA show that the effects are always positive being situated to the right of the origin with mean incremental effect of 53.4% (CI: 47.4%; 59.5%) (Figure 4). This means that there is no uncertainty that using RDT diagnosis always led to an improvement in the number of appropriately treated patients compared to presumptive diagnosis. With respect to incremental costs, there are both positive and negative values meaning that there is uncertainty in the direction of RDT *versus* presumptive costs with mean incremental cost of US$2.4 (CI: −US$7.5; US$15.7).

**Figure 5:**
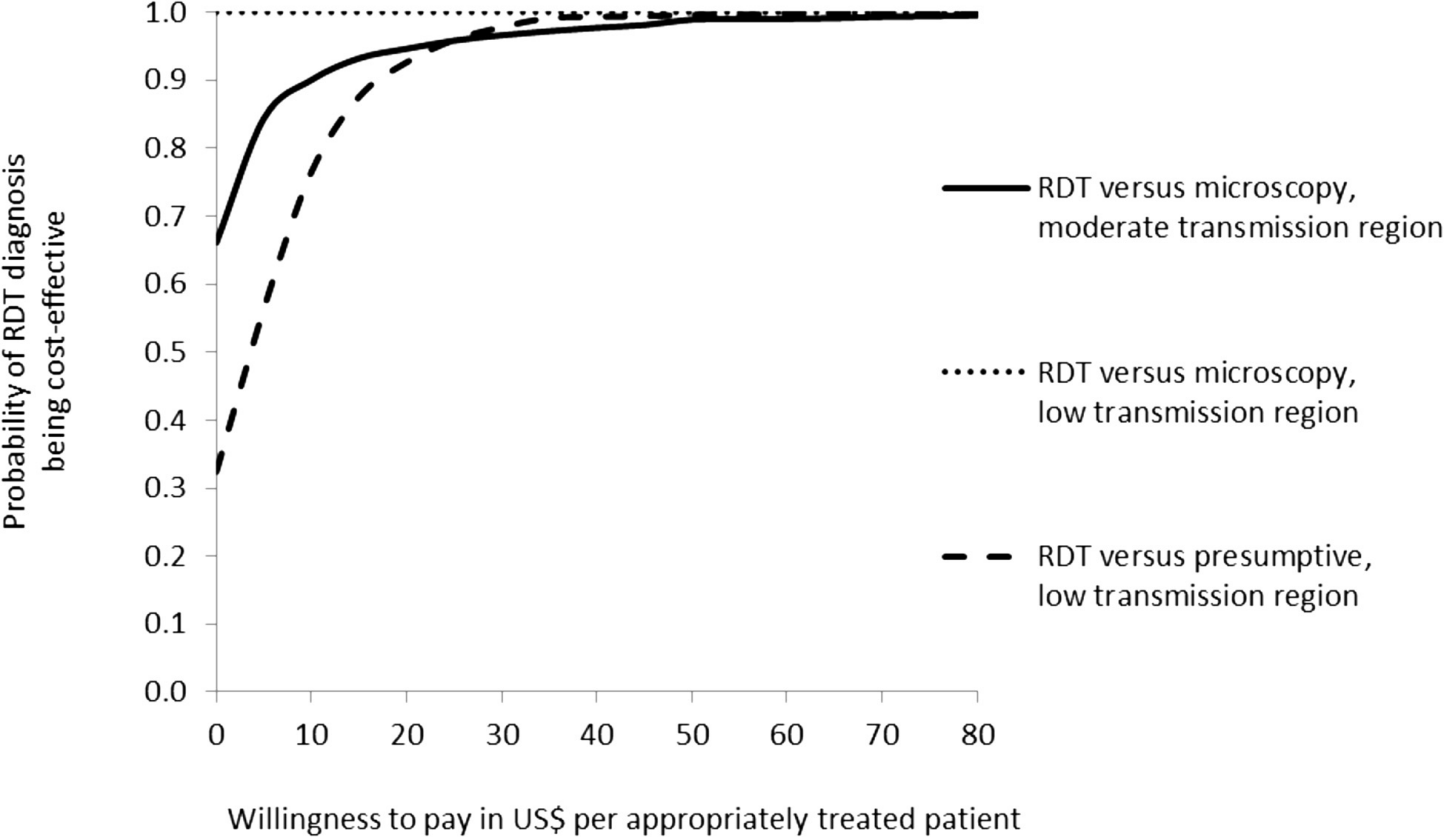
Cost-effectiveness acceptability curves for replacing microscopy diagnosis and presumptive diagnosis by RDT diagnosis in a moderate and a low transmission region of Afghanistan, 2009.

The uncertainty displayed in these scatterplots may be summarized as cost-effectiveness acceptability curves [39,40] showing the probability that the introduction of RDT diagnosis is a cost-effective intervention as a function of the decision maker’s willingness to pay (WTP) for an additional appropriately treated patient (Figure 5). In the moderate transmission area, if the WTP is US$5 per appropriately treated patient, the probability of RDT diagnosis being the cost-effective intervention is 84% increasing to 90% and above if the WTP is US$10 or higher. Comparing RDT diagnosis to microscopy in the low transmission region, there is no uncertainty that RDT diagnosis is the cost-effective intervention since the probability is 100% for any positive value of WTP up to approximately US$330 and still 99% at a WTP of US$500. Finally, introducing RDTs into health centres in the low transmission area with currently only presumptive diagnosis would be cost-effective with a probability of 56% if the WTP for an additional appropriately treated patient was US$5 increasing to 77 and 98% if the WTP was US$10 and US$30, respectively. In summary, RDT diagnosis was found to have a high probability of being a cost-effective intervention in all three settings even at relatively low levels of WTP per appropriately treated patient.

The results of the scenario analysis assessing the cost per appropriately treated malaria patient are presented in Table 5. In the low transmission setting with recently installed microscopes, the incremental cost per additional appropriately treated malaria patient after diagnosis by microscopy compared with blanket prescribing of paracetamol to fever patients was US$1,102 - three times the incremental cost of US$384 of diagnosis by RDT. The incremental cost per appropriately treated malaria patient was much lower in the moderate transmission area in both the microscopy and RDT arms. In the low transmission area where presumptive diagnosis is the norm, this analysis was not performed since no malaria cases were found.

**Table 5:** **Scenario analysis: Incremental cost-effectiveness ratio (ICER) for appropriately treated malaria patient from a health sector perspective in US$ comparing microscopy and RDT diagnostic methods with paracetamol in two regions of Afghanistan, 2009**

	**Moderate transmission region**			**Low transmission region**					
				**Area with microscopes**			**Area without microscopes**		
	**Microscopy**	**RDT**	**Paracetamol**	**Microscopy**	**RDT**	**Paracetamol**	**Clinical**	**RDT**	**Paracetamol**
Appropriately treated malaria patients (%)	21.03	21.01	0.00	0.78	0.38	0.00	0.00^&^	0.00^&^	0.00^&^
Health sector cost per patient in US$	2.57	2.55	0.48	8.94	1.85	0.38	0.50	1.84	0.38
ICER, RDT *versus* paracetamol			9.85			384.20			-
ICER, microscopy *versus* paracetamol			9.94			1101.21			-

^&^ No malaria cases.

The second scenario analysis examined replacing chloroquine with ACT to treat all malaria cases (regardless of the infecting species), and then exploring the effect of changes in the price of ACT. It was found that increasing the ACT price will lead to RDTs becoming increasingly cost-effective and eventually dominant in the low transmission area with no current parasitological test facilities. In the other two settings where RDTs are already shown to be dominant, they remained dominant as chloroquine was replaced by ACT and as the price of ACT increased.

In all study arms in both regions, average household costs constituted a significant share of societal costs (57% or above). However, the level of household cost varied widely across individuals in the sample, with the majority experiencing no or very little household cost related to the illness - 75% of participants reported having no out-of-pocket expenditure for treatment seeking and 65% of patients found that their illness prevented their normal activities for two days or less. High costs in a minority of households contributed to the high average household costs (Table 4). When household costs were excluded from the calculation to assess ICERs from a health sector perspective the cost decreased, but this did not change any of the conclusions from the main cost-effectiveness analysis. In the setting with low transmission and no parasitological diagnosis, the incremental cost from a health sector perspective per additional appropriately treated patient was US$2.5 if RDT diagnosis was introduced instead of presumptive diagnosis (Table 4).

## Discussion

These results support a strategy of introducing RDTs in all three distinct settings included in the study as it is cost-effective compared to the alternative. Most cost-effectiveness studies of RDTs have used falciparum-specific tests, while the present study used more expensive bivalent tests to provide diagnosis of both *P. falciparum* and *P. vivax.*

The sites show important differences in cost-effectiveness in different settings of malaria transmission intensity. In the moderate transmission area with long established microscopes, RDTs dominated microscopy due to a higher proportion of appropriately treated patients and, on average, at a slightly lower cost (although the difference was modest). The results of the PSA showed that there was a high probability that relying on RDTs for malaria diagnosis would be a cost-effective intervention even at relatively low values of WTP (≤US$5). In the low transmission area with newly established microscopy, RDTs had on average a smaller advantage over microscopy in terms of appropriately treated patients, but this was achieved at a much lower cost. RDTs therefore dominated microscopy and the PSA indicates that introducing RDTs would have a high probability of being the cost-effective intervention. This conclusion was driven by the high cost per microscopy-based diagnosis, which was, in turn, caused by the use of microscopes solely for the purpose of diagnosing malaria in an area with very little malaria transmission and where local elimination was the goal. This approach leaves microscopes and microscopists under-utilized. If the introduction of new microscopes had incorporated the use of a range of diagnostic tests rather than just malaria detection, the cost advantage of RDTs would diminish. The sensitivity analysis carried out which assumed that the cost per malaria slide was similarly low to that of the moderate transmission area, where microscopes have a range of functions, showed that RDTs would still dominate microscopy in the low transmission setting at the reduced microscopy cost. Finally, in the low transmission area with no access to microscopy, there was a strong case for replacing presumptive diagnosis by RDT from a clinical point of view as the proportion of appropriately treated patients in the trial increased five-fold. In this setting, RDTs had a higher cost because of their associated commodity prices, training and extra personnel time. However, because of the magnitude of the effect, the incremental cost per additional appropriately treated patient was low: US$4.5 from a societal perspective and US$2.5 from a health sector perspective. If health policy makers are willing to pay at least such an amount, then RDTs should be introduced in this area to improve patient treatment.

Average household costs were found to be a significant component of the cost of providing services in this study although the burden varied widely across households in the sample: the majority of households experienced no cost while a minority had high costs. Household costs as a proportion of total costs constituted upwards of 57% in all study arms with both out-of-pocket expenditure for healthcare seeking and opportunity cost of time lost contributing to this high cost. This was in line with similar research in Africa where household costs also contributed a high share of societal cost [24,44]. In the moderate transmission region, appropriately treated patients had on average lower household cost than inappropriately treated patients, as also found in Ghana [24]. The opposite was found in the low transmission region where inappropriately treated patients experienced the lowest household cost (Table 3), a finding that may be considered counterintuitive. One possible explanation is the primary outcome of this study defined as ‘appropriate treatment for malaria’: a patient with a parasite-positive reference slide receiving ACT/chloroquine or a patient with a negative reference slide not receiving ACT/chloroquine. Patients with true malaria correctly treated with ACT or chloroquine have a high probability of being cured and therefore have a low risk of incurring additional household cost. Contrary to this, patients with non-malarial causes of fever, as in the vast majority of cases in low transmission settings, are more difficult to diagnose and treat correctly. This could lead to more frequent and costly additional healthcare seeking by households. Since malaria prevalence was close to zero in the low transmission region, patients suffered mainly from fevers not caused by malaria where household cost may be higher.

These high household costs did not influence the conclusions of the cost-effectiveness analysis of introducing RDTs in the different settings. Leaving out all household cost and calculating ICERs from a health sector perspective still supported RDTs replacing current diagnostic techniques as the cost-effective intervention (Table 4). Furthermore, the level of household cost depends on the value assigned to lost time in this study. Sensitivity analysis using a minimum and maximum value per day of US$0.50 and US$3.00, respectively, instead of US$1.23, did not change the conclusions with respect to supporting RDTs as a cost-effective intervention in all study settings. Other studies have examined the effect of clinician compliance to the test result (including this trial). In Tanzania, poor compliance to test results reduced the cost-effectiveness of parasite-based diagnosis considerably [20]. The data used in this study includes the non-compliance to tests results by prescribers, which occurred in 10-30% of malaria negative patients. Comparative cost-effectiveness would be unlikely to change and there was little difference in compliance between RDTs and microscopy [3].

Good quality microscopy has potential added value such as the diagnosis of other conditions and clinical advantages in assessing density of infection, confirming mixed infections, and assessing cure. In the eastern region, microscopes and microscopists performed other tests, including TB microscopy, but in the northern region in microscopy health centres there were no additional benefits other than malaria diagnosis. Thus, in the eastern region health centres, only the ‘malaria-associated’ costs (time and materials) were allocated to microscopy to compare with RDTs for malaria case management whilst in northern region health centres, the entire cost of microscopes and microscopists are attributed to malaria detection. Testing for other diseases should be considered a separate intervention and thus cost and effects of testing for other diseases were excluded to make a direct comparison examining case management only.

The results indicate that RDTs are cost-effective or dominant in each setting. RDTs should therefore be used in place of microscopy, if the microscopy is used solely for malaria diagnosis. Even with multiple use microscopy, an alternative recommendation could be that the laboratory primarily relies on RDTs, with the implicit assumption that the freed capacity of the microscopy and microscopists be used for other activities.

The analyses presented here estimate the cost-effectiveness of the use of RDTs in an area of mixed malaria endemicity outside Southeast Asia and the Pacific and within a specific healthcare system and socio-economic setting. However, the effects of RDTs are likely to be generalizable to other areas of Afghanistan and the wider region. The health service setting in which the trial took place is a basic package of health services, replicated in all areas of the country. The services provided by clinics in Afghanistan and among regional neighbour countries may be seen as similar at the primary care level. Malaria endemicity can also be seen as similar across the range of malaria transmission intensity encountered in this study. While the effect of RDTs is likely to be similar, the costs of service provision and to the household are likely to differ significantly across the region. Because of these differences, assessment of cost-effectiveness would be desirable in each setting where RDTs are being considered for introduction; the decision to switch to RDTs is potentially a very expensive one.

As follow-up household costs were only collected on a sub-sample of patients, a decision analytic modelling approach was chosen. This has the benefit of facilitating scenario analysis and PSA, both of which were undertaken. The PSA enabled uncertainty around the mean parameters to be taken into account in the presentation of the results. Statistical analysis showed no covariate imbalances at enrolment requiring adjustment and allowing adjustment for clinic variation, as in the main trial results, indicated a significantly stronger effect in only one region, all other regions already showing a significant effect [3]. The results are potentially conservative, which already show RDTs to be cost-effective at low WTP thresholds and even dominant in the comparison with microscopy.

Finally, health outcomes could have been extrapolated beyond the trial period for patients in terms of cost per Disability-Adjusted Life Year (DALY). In this trial, as most patients were malaria negative, the impact on DALYs between arms would be similar unless more information had been captured about the range and severity of the non-malarial causes of fever. Not enough is known about complications from non-febrile illness but almost all studies indicate that where malaria diagnosis is introduced, use of anti-malarial treatment reduces at the expense of increased (and probably unnecessary) use of antibiotics in the majority of patients. A small group of patients, however, may genuinely require antibiotic treatment for potentially serious bacterial or parasitic infections (such as brucellosis, coxiella, rickettsial diseases, and leptospirosis). The identification of patients with such conditions using point of care diagnostics is a recognized priority for further research.

## Conclusion

In this context, introducing malaria RDTs with a standard training package is shown to be a desirable intervention on cost-effectiveness grounds. In both the moderate and low transmission areas, RDT diagnosis dominates microscopy and is therefore cost-effective. In settings currently without parasitological diagnosis, the introduction of RDTs leads to a large improvement in the proportion of patients appropriately treated at a low cost, particularly from a health sector perspective. The analyses presented in this paper suggest that the RDT intervention provides value for money in terms of appropriately treated febrile patient in each of the trial settings.

## Additional file

Additional file 1:
**Model inputs.**
