# Supplementary material for: Cost-effectiveness of malaria diagnosis using rapid diagnostic tests compared to microscopy or clinical symptoms alone in Afghanistan
Source: Malar J. 2015 May 28;14:217. doi: 10.1186/s12936-015-0696-1 (PMC4450447; doi:10.1186/s12936-015-0696-1)
Supplement: Additional file 1: — Model inputs. [file 12936_2015_696_MOESM1_ESM.docx]

**Additional file 1: Model inputs**

| **Moderate transmission region** | **RDT arm** | | | | **Microscopy arm** | | | | |  | |
| --- | --- | --- | --- | --- | --- | --- | --- | --- | --- | --- | --- |
|  | ***Deterministic*** | ***Yes*** | ***N = total*** |  | ***Deterministic*** | ***Yes*** | |  | ***N = total*** | **Distribution** | **Description** |
| Reference diagnosis Pf+ | 0.0383 | 76 | 1983 |  | 0.0350 | | 71 |  | 2028 | Deterministic | Prevalence |
| Reference diagnosis Pv+ | 0.2189 | 434 | 1983 |  | 0.1948 | | 395 |  | 2028 | Deterministic | Prevalence |
| Reference diagnosis NMFI | 0.7428 | 1473 | 1983 |  | 0.7702 | | 1562 |  | 2028 | Deterministic | Prevalence |
| Ref. diag. Pf+ and clinic test Pf+ | 0.4342 | 33 | 76 |  | 0.9296 | | 66 |  | 71 | Dirichlet | Sensitivity/specificity |
| Ref. diag. Pf+ and clinic test Pv+ | 0.2763 | 21 | 76 |  | 0.0282 | | 2 |  | 71 | Dirichlet | Sensitivity/specificity |
| Ref. diag. Pf+ and clinic test NMFI | 0.2895 | 22 | 76 |  | 0.0423 | | 3 |  | 71 | Dirichlet | Sensitivity/specificity |
| Ref. diag. Pv+ and clinic test Pf+ | 0.0115 | 5 | 434 |  | 0.0127 | | 5 |  | 395 | Dirichlet | Sensitivity/specificity |
| Ref. diag. Pv+ and clinic test Pv+ | 0.8802 | 382 | 434 |  | 0.9291 | | 367 |  | 395 | Dirichlet | Sensitivity/specificity |
| Ref. diag. Pv+ and clinic test NMFI | 0.1083 | 47 | 434 |  | 0.0582 | | 23 |  | 395 | Dirichlet | Sensitivity/specificity |
| Ref. diag. NMFI and clinic test Pf+ | 0.0095 | 14 | 1472 |  | 0.0179 | | 28 |  | 1562 | Dirichlet | Sensitivity/specificity |
| Ref. diag. NMFI and clinic test Pv+ | 0.1236 | 182 | 1472 |  | 0.0519 | | 81 |  | 1562 | Dirichlet | Sensitivity/specificity |
| Ref. diag. NMFI and clinic test NMFI | 0.8668 | 1276 | 1472 |  | 0.9302 | | 1453 |  | 1562 | Dirichlet | Sensitivity/specificity |
| **RDT arm** | | | |  | **Microscopy arm** | | | | | |  |

| **Treatment received** | **ACT** | **CQ** | **AM** | **AB no AM** | **no AM/AB** | **Total** | **ACT** | **CQ** | **AM** | **AB no AM** | **No AM/AB** | **Total** | **Distribution** |
| --- | --- | --- | --- | --- | --- | --- | --- | --- | --- | --- | --- | --- | --- |
| Ref. diag. Pf+ and clinic test Pf+ | 58 | 1 | 7 | 0 | 0 | 66 | 24 | 0 | 8 | 0 | 1 | 33 | Dirichlet |
| Ref. diag. Pf+ and clinic test Pv+ | 0 | 2 | 0 | 0 | 0 | 2 | 0 | 21 | 0 | 0 | 0 | 21 | Dirichlet |
| Ref. diag. Pf+ and clinic test NMFI | 0 | 0 | 0 | 3 | 0 | 3 | 0 | 1 | 12 | 0 | 9 | 22 | Dirichlet |
| Ref. diag. Pv+ and clinic test Pf+ | 5 | 0 | 0 | 0 | 0 | 5 | 3 | 0 | 2 | 0 | 0 | 5 | Dirichlet |
| Ref. diag. Pv+ and clinic test Pv+ | 0 | 365 | 1 | 0 | 1 | 367 | 1 | 381 | 0 | 0 | 0 | 382 | Dirichlet |
| Ref. diag. Pv+ and clinic test NMFI | 0 | 3 | 0 | 9 | 11 | 23 | 0 | 12 | 0 | 24 | 11 | 47 | Dirchlet |
| Ref. diag. NMFI and clinic test Pf+ | 23 | 1 | 3 | 0 | 1 | 28 | 9 | 0 | 4 | 0 | 1 | 14 | Dirichlet |
| Ref. diag. NMFI and clinic test Pv+ | 0 | 81 | 0 | 0 | 0 | 81 | 0 | 181 | 0 | 1 | 0 | 182 | Dirichlet |
| Ref. diag. NMFI and clinic test NMFI | 0 | 184 | 0 | 781 | 488 | 1453 | 0 | 184 | 0 | 693 | 399 | 1276 | Dirichlet |

|  |  |  | |  |  |  | | |  |  |
| --- | --- | --- | --- | --- | --- | --- | --- | --- | --- | --- |
| **Low transmission region** | **RDT arm** | | |  | **Microscopy arm** | | | |  | |
|  | ***Deterministic*** | ***Yes*** | ***N = total*** |  | ***Deterministic*** | ***Yes*** |  | ***N = total*** | **Distribution** | **Description** |
| Reference diagnosis Pv+ | 0.0097 | 5 | 515 |  | 0.0038 | 2 |  | 523 | Deterministic | Prevalence |
| Reference diagnosis NMFI | 0.9903 | 510 | 515 |  | 0.9962 | 521 |  | 523 | Deterministic | Prevalence |
| Ref. diag. Pv+ and clinic test Pv+ | 0.8000 | 4 | 5 |  | 0.0000 | 0 |  | 2 | Beta | Sensitivity/specificity |
| Ref. diag. Pv+ and clinic test NMFI | 0.2000 | 1 | 5 |  | 1.0000 | 2 |  | 2 | Beta | Sensitivity/specificity |
| Ref. diag. NMFI and clinic test Pv+ | *Missing complete data* | |  |  | 0.0115 | 6 |  | 521 | Beta | Sensitivity/specificity |
| Ref. diag. NMFI and clinic test NMFI | *Missing complete data* | |  |  | 0.9885 | 515 |  | 521 | Beta | Sensitivity/specificity |
| **RDT arm** | | |  |  | **Microscopy arm** | | | |  |  |

| **Treatment received** | **ACT** | **CQ** | **AM** | **AB no AM** | **no AM/AB** | **Total** | **ACT** | **CQ** | **AM** | **AB no AM** | **no AM/AB** | **Total** | **Distribution** |
| --- | --- | --- | --- | --- | --- | --- | --- | --- | --- | --- | --- | --- | --- |
| Ref. diag. Pv+ and clinic test Pv+ | 0 | 0 | 0 | 0 | 0 | 0 | 0 | 4 | 0 | 0 | 0 | 4 | Dirichlet |
| Ref. diag. Pv+ and clinic test NMFI | 0 | 2 | 0 | 0 | 0 | 2 | 0 | 0 | 0 | 0 | 1 | 1 | Dirichlet |
| Ref. diag. NMFI and clinic test Pv+ | 0 | 6 | 0 | 0 | 0 | 6 | 0 | 121 | 0 | 389 | 0 | 510 | Dirichlet |
| Ref. diag. NMFI and clinic test NMFI | 0 | 97 | 0 | 367 | 51 | 515 | 0 | 0 | 0 | 0 | 0 | 0 | Dirichlet |

| **Low transmission region** |  |  | | |  | |  |  | | | | | |  |  |
| --- | --- | --- | --- | --- | --- | --- | --- | --- | --- | --- | --- | --- | --- | --- | --- |
|  | **RDT arm** | | | | | | **Clinical diagnosis arm** | | | | | | | |  |
|  | ***Deterministic*** | | ***Yes*** | ***N= total*** | |  | ***Deterministic*** | | ***Yes*** | |  | | ***N = total*** | **Distribution** | **Description** |
| Reference diagnosis Pv+ | 0.0000 | | 0 | 321 | |  | 0.0000 | | 0 | |  | | 325 | Deterministic | Prevalence |
| Reference diagnosis NMFI | 1.0000 | | 321 | 321 | |  | 1.0000 | | 325 | | |  | 325 | Deterministic | Prevalence |
| Ref. diag. NMFI and clinic test Pv+ | 0.9938 | | 319 | 321 | |  | 0.0031 | | 1 |  | | | 325 | Beta | Sensitivity/specificity |
| Ref. diag. NMFI and clinic test NMFI | 0.0062 | | 2 | 321 | |  | 0.9969 | | 324 |  | | | 325 | Beta | Sensitivity/specificity |
| **RDT arm** | | | | | | | **Clinical diagnosis arm** | | | | | | | |  |

| **Treatment received** | **ACT** | **CQ** | **AM** | **AB no AM** | **no AM/AB** | **Total** | **ACT** | **CQ** | **AM** | **AB no AM** | **no AM/AB** | **Totals** | **Distribution** |
| --- | --- | --- | --- | --- | --- | --- | --- | --- | --- | --- | --- | --- | --- |
| Ref. diag. NMFI and clinic test Pv+ | 0 | 1 | 0 | 0 | 0 | 1 | 0 | 276 | 3 | 31 | 9 | 319 | Dirchlet |
| Ref. diag. NMFI and clinic test NMFI | 0 | 107 | 2 | 171 | 41 | 321 | 0 | 2 | 0 | 0 | 0 | 2 | Dirichlet |

|  | | |  |  |  |  | |  |  |
| --- | --- | --- | --- | --- | --- | --- | --- | --- | --- |
| **Costs per patient (US$ unless specified as days)** | | |  |  |  |  | |  |  |
| **Moderate transmission region** | ***Mean (sd)*** | **Shape** | ***Scale*** |  | **Distribution** |  | |  |  |
| Out of pocket appropriate treatment | 3.77 (12.77) | 0.0872 | 43.2295 |  | Gamma |  |  | |  |
| Lost time (days) appropriate treatment | 1.38 (3.05) | 0.2058 | 6.7242 |  | Gamma |  |  | |  |
| Carer lost time (days) appropriate treatment | 0.53 (1.70) | 0.0969 | 5.4696 |  | Gamma |  |  | |  |
| Out of pocket inappropriate treatment | 6.92 (19.63) | 0.1242 | 55.6967 |  | Gamma |  |  | |  |
| Lost time (days) inappropriate treatment | 2.16 (3.51) | 0.3798 | 5.6998 |  | Gamma |  |  | |  |
| Carer lost time (days) inappropriate treatment | 0.79 (1.62) | 0.2397 | 3.3006 |  | Gamma |  |  | |  |
|  |  |  |  |  |  |  |  | |  |
| **Low transmission region** | ***Mean (sd)*** | **Shape** | ***Scale*** |  | **Distribution** |  |  | |  |
| Out of pocket appropriate treatment | 3.12 (6.86) | 0.2068 | 15.095 |  | Gamma |  |  | |  |
| Lost time (days) appropriate treatment | 4.69 (3.85) | 1.4892 | 3.1523 |  | Gamma |  |  | |  |
| Carer lost time (days) appropriate treatment | 2.60 (2.46) | 1.1144 | 2.3287 |  | Gamma |  |  | |  |
| Out of pocket inappropriate treatment | 2.26 (4.80) | 0.222 | 10.1959 |  | Gamma |  |  | |  |
| Lost time (days) inappropriate treatment | 4.21 (2.65) | 2.5207 | 1.6713 |  | Gamma |  |  | |  |
| Carer lost time (days) inappropriate treatment  Unit cost per outpatient visit, microscopy, RDT and drugs in Tables 2a and 2b | 2.21(1.82) | 1.483 | 1.4921 |  | Gamma |  |  | |  |

**Treatment for those patients who received both an antimalarial and an antibiotic.**

| **Moderate transmission region** |  | **RDT arm** | | |  | **Microscopy arm** | | | | |  |
| --- | --- | --- | --- | --- | --- | --- | --- | --- | --- | --- | --- |
|  | ***Deterministic*** | | ***Yes*** | ***N = total*** |  | ***Deterministic*** | ***Yes*** |  | ***N = total*** | **Distribution** | **Description** |
| Ref. diag. Pf+ and clinic test Pf+ and ACT/AB | - |  | |  |  | 0.0172 | 1 |  | 58 | Beta | Treatment |
| Ref. diag. Pf+ and clinic test Pv+ and CQ/AB | 0.0476 | 1 | | 21 |  | - |  |  |  | Beta | Treatment |
| Ref. diag. Pv+ and clinic test Pv+ and CQ/AB | 0.0210 | 8 | | 381 |  | 0.0384 | 14 |  | 365 | Beta | Treatment |
| Ref. diag. Pv+ and clinic test NMFI and CQ/AB | 0.0833 | 1 | | 12 |  | - |  |  |  | Beta | Treatment |
| Ref. diag. NMFI and clinic test Pf+ and ACT/AB | - |  | |  |  | 0.0435 | 1 |  | 23 | Beta | Treatment |
| Ref. diag. NMFI and clinic test Pv+ and CQ/AB | 0.0387 | 7 | | 181 |  | 0.0370 | 3 |  | 81 | Beta | Treatment |
| Ref. diag. NMFI and clinic test NMFI and CQ/AB | 0.0435 | 8 | | 184 |  | 0.0598 | 11 |  | 184 | Beta | Treatment |
| **Low transmission region** |  | | |  |  |  |  |  |  |  |  |
| Ref. diag. Pv+ and clinic test NMFI and CQ/AB | *-* | | |  |  | 1.0000 | 2 |  | 2 | Beta | Treatment |
| Ref. diag. NMFI and clinic test Pv+ and CQ/AB | 0.4583 | 55 | | 120 |  | 0.1667 | 1 |  | 6 | Beta | Treatment |
| Ref. diag. NMFI and clinic test NMFI and CQ/AB | - |  | |  |  | 0.4433 | 43 |  | 97 | Beta | Treatment |
| **Low transmission region** |  |  | |  |  |  |  |  |  |  |  |
| Ref. diag. NMFI and clinic test Pv+ and CQ/AB | 0.0543 | 15 | | 276 |  | - |  |  |  | Beta | Treatment |
| Ref. diag. NMFI and clinic test NMFI and CQ/AB |  |  | |  |  | 0.1215 | 13 |  | 107 | Beta | Treatment |
| Ref. diag. NMFI and clinic test NMFI and  Other AM/AB |  |  | |  |  | 0.2000 | 1 |  | 5 | Beta | Treatment |

Pf: Plasmodium falcipium, Pv: Plasmodium vivax, NMFI: non-malarial febrile illness, ACT: artemisinin-based combination therapy, CQ: quinine, AM: antimalarial, AB: antibiotic.
